# Another Case of Saturnine Encephalopathy

**Published:** 1907-09-28

**Authors:** 


### Illustrative Cases.

### Another Case of Saturnine Encephalopathy.

The following case illustrates saturnine encephalopathy, in which, instead of the more common coma and epileptiform convulsions, the symptoms bordered more upon those of temporary insanity—mania with delusions. If the patient had not been known to have lived very temperately his trouble might well have been attributed to the effects of drink.

He was a white-lead cooper, aged 38, who had always been a healthy man, almost teetotal; he was accustomed to do a good and regular day's work, and had never suffered from anything but an occasional cold. There was no history of any neurosis or insanity in the family. The patient had never had syphilis. Five months previous to the present trouble he had acute abdominal colic, lasting a few days, but cured by castor oil and opium. There had been no recurrence until a day or two ago, when constipation began to trouble him badly, and it was soon followed by acute abdominal colic. He then remembered that he had noticed that his hands had " become peculiar " during the last two weeks, so that he could not extend them properly.

When seen he was noticed to be a well-developed, dark-complexioned man, in the prime of life; his face was pale, and expressive of pain; every now and then he writhed about in bed, groaning in the paroxysm of colic, with his hands pressed to the front and sides of his abdomen. Although he could use his hands in this way they were affected by the typical wrist-drop. The bowels had not been opened for four days.

The teeth were in very bad condition, covered with tartar, and there was an irregular blue line near the free edge of the gums.

The temperature was 98.4, pulse rate 88, and respiration rate 20 per minute.

Examination of the abdomen was difficult, but nothing organically wrong could be made out. The heart and lungs were natural. The urine contained neither albumin nor sugar.

The case seemed to be a straightforward example of chronic lead-poisoning; castor oil and tincture of opium were administered in a single dose, and potassium iodide was given three times a day.

During the night it was noticed that the patient's mental state did not seem quite sound; he talked rubbish, and tried continually to get out of bed. He hardly slept at all. The next day he seemed more sensible, but the following night he was much worse again, calling out at the top of his voice, swearing at everyone who came near him, and using the most filthy language without the least provocation. The bowels having been well opened the colic diminished and disappeared, but the mental trouble persisted for seven days and nights. He became violent, striking both himself and his relations, upsetting the wash-stand and other furniture, breaking everything within his reach to pieces. He required the most careful watching to prevent him doing himself grievous bodily harm. He took to banging his head with great force against the bed-posts, and presently he got the idea that four men had come through the window during the night, and had knocked him about in a ruffianly manner. He wove great detail into this story as time went on, constantly repeating it with additions, amongst which was the idea that he had unsuccessfully tried to bribe the villains to leave him alone, with the sum of one and ninepence.

When this state of affairs had continued for nearly a week the mental symptoms got better fairly rapidly, and throughout the period of recovery from the peripheral neuritis and other toxic effects of the lead, there was no sign of saturnine encephalopathy, and the patient has remained quite well since.

